# Supplementary material for: General practitioners’ consultation counts and associated factors in Swiss primary care – A retrospective observational study
Source: PLoS One. 2019 Dec 31;14(12):e0227280. doi: 10.1371/journal.pone.0227280 (PMC6938322; doi:10.1371/journal.pone.0227280)
Supplement: Attachment — Submitted filename: Response to Reviewers.docx [file pone.0227280.s001.docx]

**Response to Reviewers**

**Reviewer #1:**

Dear Authors,

thanks a lot for your precious work in this field of primary care where data like this is scare. I do have some minor comments and questions for you to be answered.

- *Answer: Thank you very much to Reviewer #1 for this motivating feedback!*

Abstract:

line 40: Instead of "not including" I would rather us the wording - "Exclusion of..."

- *Answer: Done*

line 46-48: Please consider choosing a more positive formulation rather "not affect.... negatively".

- *Answer: Since we refute the “fear” of a decreased efficiency for these GPs, we would like to keep the (“negative”) meaning. We however rephrased it.*

Methods:

line 108: What do you mean by "a spatial typology scheme"?

- *Answer: The Swiss Federal Statistical Office categorizes districts based on the zip code. We rephrased the sentence forgoing the term “spatial typology scheme”.*

line 127: Please decide whether you "covariates" or "co-variates" throughout the manuscript.

- *Answer: “Co-variates” is due to hyphenation at line break, we used “covariates” throughout the manuscript.*

Results:

line 162-164: Did you correct for half-days in this low part-time workers? 30% might mean 3 half days of work (while the kids are in kindergarten) - which might explain why they only see 50% of the patients a full-time GP sees.

- *Answer: We completely agree that these might be half days of work (cf. line 224). This is why we use total yearly consultation counts in the regression.*

also line 265: Please explain why

- *Answer: (Assuming that you are referring to line 165) We did not address this because it is pure speculation, but it is conceivable that full-time workers, being more often self-employed, spend more time on administrative tasks and thus hold less consultations per day.*

table 1 - table 2: How do you explain the difference in median age of patients (73 vs. a maximum of 63 years in 90% part-time workers and even lower in all other groups?

- *Answer: The number “73” in table 2 refers to the change in consultation counts per one year increase in the median age of the GP’s patient population (the unit is consultations, not years).*

Looking forward to your reply.

**Reviewer #2:**

This study is an interesting and well written study.

Just one remark. In the discussion section: the sub-tittle "summary" should be deleted because it induces a misunderstanding.

- *Answer: Many thanks also to Reviewer #2 for this positive feedback! We deleted the sub-title “summary” as per your request.*
